# Flavan‐3‐ol Microbial Metabolites Modulate Proteolysis in Neuronal Cells Reducing Amyloid‐beta (1‐42) Levels

**DOI:** 10.1002/mnfr.202100380

**Published:** 2021-08-07

**Authors:** Valentina Cecarini, Massimiliano Cuccioloni, Yadong Zheng, Laura Bonfili, Chunmei Gong, Mauro Angeletti, Pedro Mena, Daniele Del Rio, Anna Maria Eleuteri

**Affiliations:** ^1^ School of Biosciences and Veterinary Medicine University of Camerino Camerino Italy; ^2^ Human Nutrition Unit Department of Food and Drugs University of Parma Parma Italy; ^3^ Microbiome Research Hub University of Parma Parma Italy

**Keywords:** Alzheimer's disease, amyloid, autophagy, polyphenol metabolites, proteasome

## Abstract

**Introduction:**

Alzheimer's disease (AD) is a progressive neurodegeneration characterized by extensive protein aggregation and deposition in the brain, associated with defective proteasomal and autophagic‐lysosomal proteolytic pathways. Since current drugs can only reduce specific symptoms, the identification of novel treatments is a major concern in AD research. Among natural compounds, (poly)phenols and their derivatives/metabolites are emerging as candidates in AD prevention due to their multiple beneficial effects. This study aims to investigate the ability of a selection of phenyl‐γ‐valerolactones, gut microbiota‐derived metabolites of flavan‐3‐ols, to modulate the functionality of cellular proteolytic pathways.

**Methods and Results:**

Neuronal SH‐SY5Y cells transfected with either the wild‐type or the 717 valine‐to‐glycine amyloid precursor protein mutated gene are used as an AD model and treated with 5‐(4ʹ‐hydroxyphenyl)‐γ‐valerolactone, 5‐(3ʹ,4ʹ‐dihydroxyphenyl)‐γ‐valerolactone and 5‐(3ʹ‐hydroxyphenyl)‐γ‐valerolactone‐4ʹ‐sulfate. Combining in vitro and in silico studies, it is observed that the phenyl‐γ‐valerolactones of interest modulated cellular proteolysis via proteasome inhibition and consequent autophagy upregulation and inhibited cathepsin B activity, eventually reducing the amount of intra‐ and extracellular amyloid‐beta (1‐42) peptides.

**Conclusion:**

The findings of this study establish, for the first time, that these metabolites exert a neuroprotective activity by regulating intracellular proteolysis and confirm the role of autophagy and cathepsin B as possible targets of AD preventive/therapeutic strategies.

## Graphical abstract

Neuronal SH‐SY5Y cells treatment with phenyl‐γ‐valerolactones, gut microbiota‐derived metabolites of flavan‐3‐ols, results in intracellular proteolysis modulation. In detail, cells exhibit inhibition of the functionality of the proteasomal system and the simultaneous activation of the autophagic process. In addition, treated cells show a compromised cathepsin B activity. The fine regulation of these proteolytic systems finally results in the decrease of both intra‐ and extracellular Aβ(1‐42) peptides in neurons.

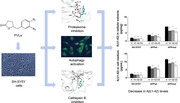

## 1 Introduction

Alzheimer's disease (AD) is a neurodegenerative condition mostly characterized by extensive protein aggregation with amyloid‐beta deposition in senile plaques and tau aggregation in neurofibrillary tangles. The sequential cleavage of the amyloid precursor protein (APP) through the so‐called amyloidogenic pathway is responsible for the release of amyloid‐beta (Aβ) peptides. A large part of these proteins are fragments of 40 amino acid residues in length, Aβ(1‐40), whereas a remaining portion is a 42‐residues protein, Aβ(1‐42), known as the most toxic and particularly prone to aggregation.^[^ ^1^ ^]^ Defective proteolytic pathways, oxidative and inflammatory processes further exacerbate AD pathology.^[^ ^2^, ^3^ ^]^ The ubiquitin proteasome system (UPS) and the autophagy‐lysosomal pathway are the two major catabolic pathways in eukaryotic cells. The UPS is in charge of the degradation of cytosolic and nuclear proteins, including short‐lived proteins, whereas autophagy is responsible for the clearance of protein aggregates and damaged organelles. Due to an age‐dependent decline in their activity, aberrant proteins accumulate, contributing to the onset and development of age‐related disorders, such as AD.^[^ ^4^ ^]^

A growing number of studies has associated the intake of foods rich in (poly)phenols with a reduced risk of developing neurodegenerative diseases, including AD. Dietary products such as green tea, apples, berries, cocoa, and chocolate are extremely rich in flavan‐3‐ols, a common subclass of flavonoids, and their consumption was associated with healthy brain aging, neuronal protection against disorders, and ameliorated cognitive function.^[^ ^5^, ^6^, ^7^ ^]^ Flavan‐3‐ols exist either as simple monomers called catechins or as oligomeric and complex polymeric structures known as proanthocyanidins.^[^ ^8^ ^]^ Only a small fraction of these molecules is absorbed in the upper part of the intestine, whereas most of the ingested flavan‐3‐ols are transformed by the colonic microbiota into low molecular phenolic compounds, mainly phenyl‐γ‐valerolactones (PVLs) and their related phenylvaleric acids, and subsequently conjugated to glucuronide and sulfate groups in the liver.^[^ ^9^ ^]^ Recently, increasing attention has been directed to the possible biological effects exerted by this class of molecules, being them one of the most abundant phenolic metabolites in the circulation upon consumption of a (poly)phenol‐rich diet, but still few data are available in the literature.^[^ ^8^, ^10^ ^]^ On this regard, it was demonstrated that PVLs, particularly (R)‐5‐(3ʹ,4ʹ‐dihydroxyphenyl)‐γ‐valerolactone, and their sulfated forms, protect brown adipocytes from increased production of reactive oxygen species.^[^ ^9^ ^]^ 5‐(3ʹ,5ʹ‐dihydroxyphenyl)‐γ‐valerolactone, an epigallocatechin gallate‐derived microbial metabolite, significantly induced neurites outgrowth and elongation in human neuroblastoma SH‐SY5Y cells.^[^ ^11^ ^]^ In addition, given the neuroprotective role of flavonoids, several studies investigated also the ability of these molecules and their derivatives to cross the blood brain barrier (BBB). The metabolite 5‐(3ʹ,5ʹ‐dihydroxyphenyl)‐γ‐valerolactone showed a slightly higher BBB permeability than its parental compound epigallocatechin‐3‐gallate (EGCG).^[^ ^11^ ^]^ Similarly, Angelino et al. recently evidenced that the 5‐(hydroxyphenyl)‐γ‐valerolactone‐sulfate (3ʹ,4ʹ isomer), another key microbial metabolite of flavan‐3‐ols, is able to reach the brain.^[^ ^12^ ^]^

In this study, human neuroblastoma SH‐SY5Y cells stably transfected with either the wild‐type amyloid precursor protein gene (APPwt) or the 717 valine‐to‐glycine AβPP‐mutated gene (APPmut) were treated with 5‐(4ʹ‐hydroxyphenyl)‐γ‐valerolactone, 5‐(3ʹ,4ʹ‐dihydroxyphenyl)‐γ‐valerolactone, and the conjugated form 5‐(3ʹ‐dihydroxyphenyl)‐γ‐valerolactone‐4ʹ‐sulfate. Interestingly, the mutation in the APP gene sequence is associated with familial forms of AD and promotes the increased brain accumulation of Aβ(1‐42/43) resulting in the enhancement of amyloid fibril formation from soluble Aβ,^[^ ^13^ ^]^ making these cells a suitable model to investigate the molecular mechanisms involved in AD. Here, we explored if PVLs could modulate the functionality of cellular proteolytic pathways, dissecting both proteasomal and autophagy functionality, and how the presence of the wild‐type or mutated APP form can influence the final effect. In silico analyses were performed to better elucidate the interaction between the considered metabolites and proteasome catalytic subunits and cathepsin B. In addition, the effect of proteolysis regulation on Aβ(1‐42) production and release by neuronal cells was investigated.

## 2 Experimental Section

### 2.1 Reagents and Chemicals

**Figure 1:**
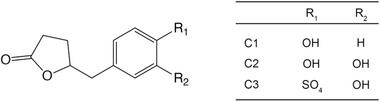
Structure of the PVLs used in this study. C1, 5‐(4ʹ‐hydroxyphenyl)‐γ‐valerolactone; C2, 5‐(3ʹ,4ʹ‐dihydroxyphenyl)‐γ‐valerolactone; C3, 5‐(3ʹ‐hydroxyphenyl)‐γ‐valerolactone‐4ʹ‐sulfate.

5‐(4ʹ‐hydroxyphenyl)‐γ‐valerolactone (C1), 5‐(3ʹ,4ʹ‐dihydroxyphenyl)‐γ‐valerolactone (C2) and 5‐(3ʹ‐hydroxyphenyl)‐γ‐valerolactone‐4ʹ‐sulfate (C3) (**Figure** **1**) were synthesized and kindly provided by Prof. C. Curti (University of Parma, Italy)^.[^ ^14^, ^15^ ^]^ The substrates Suc‐Leu‐Leu‐Val‐Tyr‐AMC, Z‐Leu‐Ser‐Thr‐Arg‐AMC, Z‐Leu‐Leu‐Glu‐AMC for assaying the chymotrypsin‐like (ChT‐L), trypsin‐like (T‐L), and peptidyl glutamyl‐peptide hydrolyzing (PGPH) activities of the proteasomal complex were purchased from Sigma‐Aldrich S.r.L. (Milano, Italy). The substrate Z‐Gly‐Pro‐Ala‐Leu‐Ala‐MCA to test the branched chain amino acids preferring (BrAAP) activity was obtained from Biomatik (Cambridge, Ontario). Aminopeptidase N (EC 3.4.11.2) for the coupled assay utilized to detect BrAAP activity was purified from pig kidney as reported elsewhere.^[^ ^16^ ^]^ Membranes for western blot analyses were purchased from Millipore (Milan, Italy). Proteins immobilized on films were detected with the enhanced chemiluminescence (ECL) system (Amersham Pharmacia Biotech, Milan, Italy). All chemicals and solvents were of the highest analytical grade available.

### 2.2 Molecular Docking

The molecular models of complexes between the PVLs and the catalytic subunits of human proteasomes were obtained according to flexible ligand‐receptor docking using Autodock 4.^[^ ^17^ ^]^ 3D structures of the molecules of interest were built and optimized with Avogadro^[^ ^18^ ^]^ and docked onto crystallographic structures of human constitutive (PDB ID: 6rgq^[^ ^19^ ^]^) and immuno‐ proteasomes (PDB ID: 6e5b^[^ ^20^ ^]^) retrieved from the RCBS Protein Data Bank.^[^ ^21^ ^]^ Specifically, a grid box (20Í20Í20 Å) was individually placed around the accessible internal portions of catalytic subunits β1, β2 and β5 and β1i, β2i and β5i immune counterparts, respectively, each box being centered on the catalytic Thr‐1 residues and covering the entire surface extending 10 Å in each direction. Likewise, the valerolactones‐cathepsin B models were obtained. The grid box was placed around the catalytic site of 3D structure of human cathepsin B (PDB ID: 1csb^[^ ^22^ ^]^), spanning 10 Å in each direction around the catalytic Cys‐29. Unless stated differently, default settings were used throughout. Resulting models were rendered with PyMOL (The PyMOL Molecular Graphics System, Version 2.4 Schrödinger, LLC).

### 2.3 Measurements of Isolated 20S Proteasome Activity

PVLs effects on the 20S constitutive and immunoproteasome peptidase activities were measured through in vitro assays performed with fluorogenic peptides as substrates. Isolation and purification of the 20S proteasome from bovine brain and thymus were performed as previously reported.^[^ ^23^ ^]^ The incubation mixture contained the compound at concentrations ranging from 0.0 to 10 µM, 1 µg of the isolated 20S proteasome, the appropriate substrate (5 µM final concentration), and 50 mm Tris/HCl (pH 8.0), up to a final volume of 100 µL. Purified proteasomes were always preincubated with each compound at 37°C for 30 min before the fluorogenic peptide substrates were added to the reaction mixture. Final incubation was performed at 37°C, and after 60 min the fluorescence of the hydrolyzed 7‐amino‐4‐methyl‐coumarin (AMC) and 4‐aminobenzoic acid (pAB) was detected (AMC, λ_exc_ = 365 nm, λ_em_ = 449 nm; pAB, λ_exc_ = 304 nm, λ_em_ = 664 nm) on a SpectraMax Gemini XPS microplate reader.

### 2.4 Cell Culture and Transfections

SH‐SY5Y cells were cultured in 1:1 Dulbecco's modified Eagle's medium and Nutrient Mixture F12 containing 10% fetal bovine serum (FBS), 2 mM glutamine, 100 units mL^−1^ penicillin, and 100 µg mL^−1^ streptomycin at 37°C in a 5% CO_2_‐containing atmosphere. The SH‐SY5Y cells stable transfection with wild type AβPP 751 (APPwt) and AβPP (Val717Gly) mutations (APPmut) was prepared as described elsewhere.^[^ ^24^ ^]^ These cells were a kind gift of Prof. Daniela Uberti from the University of Brescia. Stably transfected cells expressing either the APPwt or the APPmut construct were maintained in SH‐SY5Y medium added with G418 at a final concentration of 600 µg mL^−1^.

### 2.5 Cell Treatment and Cytotoxicity Assay

Cell viability was evaluated with the 3‐(4,5‐dimethylthiazol‐2‐yl)‐2,5‐diphenyltetrazolium bromide assay (MTT).^[^ ^25^ ^]^ Upon 6 h and 24 h treatment with increasing concentrations (0‐10 µM) of C1, C2, and C3 dissolved in DMSO, cells were washed in PBS, pH 7.5, and then MTT (final concentration 0.5 mg mL^−1^) was added to the culture medium without FBS and incubated for 2 h at 37 °C. The medium was then removed and replaced with 100 µL of DMSO. The optical density was measured at 550 nm in a microtiter plate reader. At least six cultures were utilized for each time point. Neuroblastoma cells, control and transfected cells, were then treated with C1, C2, and C3 for 6 and 24 h at the concentrations 0–1–5 µM. Compounds were dissolved in DMSO and then added to cell culture media. Control cells were included in each time point. After removing the medium and washing with cold phosphate buffered saline (PBS), cells were harvested in 4 mL of PBS and centrifuged at 1600 × g for 5 min. The pellet was resuspended in lysis buffer (20 mM Tris, pH 7.4, 250 mM sucrose, 1 mM EDTA, and 5 mM β‐mercaptoethanol) and passed through a 29‐gauge needle at least ten times. Lysates were centrifuged at 12 000×g for 15 min and the supernatants were stored at ‐80°C until use. Protein concentration was determined by the method of Bradford using bovine serum albumin (BSA) as standard.^[^ ^26^ ^]^

### 2.6 Proteasome Activity

The effects on the proteasome system were evaluated through fluorimetric assays, as previously reported,^[^ ^27^ ^]^ using the following synthetic substrates: Leu‐Leu‐Val‐Tyr‐AMC for ChT‐L, Leu‐Ser‐Thr‐Arg‐AMC for T‐L, Leu‐Leu‐Glu‐AMC for PGPH, and Gly‐Pro‐Ala‐Leu‐Ala‐AMC for BrAAP, whose test is performed with the addition of the aminopeptidase‐N (AP‐N). The incubation mixture contained 1 µg of cell lysate, the appropriate substrate, and 50 mM Tris/HCl pH 8.0, up to a final volume of 100 µL. Incubation was performed at 37 °C, and after 60 min, the fluorescence of the hydrolyzed 7‐amino‐4‐methyl‐coumarin (AMC) was recorded (AMC, λ_exc_ = 365 nm, λ_em_ = 449 nm) on a SpectraMax Gemini XPS microplate reader. The 26S proteasome ChT‐L activity was tested using Suc‐Leu‐Leu‐Val‐Tyr‐AMC as substrate and 50 mM Tris/HCl pH 8.0 buffer containing 10 mM MgCl_2_, 1 mM dithiothreitol, and 2 mM ATP. The effective 20S proteasome contribution to short peptide cleavage was evaluated with control experiments performed using specific proteasome inhibitors, Z‐Gly‐Pro‐Phe‐Leu‐CHO and lactacystin (5 µM in the reaction mixture). The fluorescence values of lysates were subtracted of the values of control assays in the presence of the two inhibitors.

### 2.7 Cathepsin B Activity

Cathepsin B proteolytic activity was measured using the fluorogenic peptide Z‐Arg‐Arg‐AMC at a final concentration of 50 µM, as previously described.^[^ ^24^ ^]^ The mixture for cathepsin B, containing 1 µg of cell lysate, was pre‐incubated in 100 mM phosphate buffer pH 6.0, 1 mM EDTA, and 2 mM dithiothreitol for 5 min at 30°C. Upon the addition of the substrate, the mixture was incubated for 15 min at 30°C. The fluorescent signal released by the hydrolyzed 7‐amino‐4‐methyl‐coumarin (AMC, λ_exc_ = 365 nm, λ_em_ = 449 nm) was detected on a SpectraMax Gemini XPS microplate reader.

### 2.8 Western Blotting Analysis

Proteins were resolved on SDS–PAGE and electroblotted onto PVDF membranes. Membranes with transferred proteins were incubated with the primary monoclonal antibody and successively with the specific peroxidase conjugated secondary antibody. Monoclonal antibodies against Ub and p27 were obtained from Santa Cruz Biotechnology, Inc. (Heidelberg, Germany). SQSTM1/p62 (sequestosome 1, herein p62) mouse monoclonal antibody was from Sigma‐Aldrich S.r.L. (Milano, Italy) and the anti‐LC3B antibody was purchased from Cell Signaling Technology, Inc. The immunoblot detection was performed with ECL Western blotting detection reagents using a ChemiDoc MP system. Each gel was loaded with molecular weight markers in the range of 12 to 225 kDa (GE Healthcare). Glyceraldehyde‐3‐phosphate dehydrogenase (GAPDH) was utilized as a control for equal protein loading: membranes were stripped and re‐probed with anti‐GAPDH monoclonal antibody (Santa Cruz Biotechnology, Heidelberg, Germany). Stripping buffer contained 200 mM glycine, 0.1% SDS, and 1% Tween 20. Immunoblot images were quantified using ImageJ 1.52p software (NIH, USA).

### 2.9 Monodansylcadaverine Assay

Upon treatment with PVLs, the formation of autophagic vacuoles was monitored with monodansylcadaverine assay (MDC, Sigma‐Aldrich S.r.L. Milano, Italy). In detail, 1 µM MDC was added to cell medium. After 10 min incubation at 37 °C, cells were washed three times with phosphate buffered solution (PBS) and immediately analyzed with a fluorescence microscope (Olympus IX71).

### 2.10 Quantification of Aβ(1‐42)

Levels of Aβ(1‐42) secreted into the medium and present in cellular extracts were determined after PVLs treatment using the Human Aβ42 solid‐phase sandwich ELISA Kit from Invitrogen, following the manufacturer's instructions. For Aβ quantification in cell medium, culture medium was collected after neuronal cells treatment, centrifuged at 300 × g for 10 min to remove non adherent cells and debris and then treated with protease inhibitors.

### 2.11 Statistical Analysis

Data are expressed as mean values ± S.D. Statistical analysis was performed with one way ANOVA, followed by the Bonferroni post hoc test using Sigma‐stat 3.1 software (SPSS, Chicago, IL, USA) and *p* < 0.05 was considered statistically significant.

## 3 Results

### 3.1 Effects of PVLs on Isolated Proteasomes Activity

PVLs were first tested on isolated constitutive and immunoproteasomes purified from bovine brain and thymus, respectively. The constitutive 20S proteasome is a barrel‐shaped complex of four stacked rings, made of seven α subunits in the two outer rings and seven β subunits in the two inner rings, the latter bearing the catalytically active sites in the β1, β2, and β5 subunits. Treatment with inflammatory cytokines induces the transcription of three additional active subunits, known as β1i, β2i, and β5i, that replace constitutive homologues during proteasome assembly.^[^ ^28^ ^]^ Proteasome proteolytic activities depend on the hydroxyl group of the N‐terminal threonine (Thr‐1) residue, responsible for cleaving peptides through a nucleophilic attack.^[^ ^29^ ^]^

Increasing concentrations of the PVLs (0‐10 µM in DMSO) were used in the fluorescent assays, as described in materials and methods. An evident inhibitory effect on the catalytic components of the two enzymes, namely the ChT‐L (associated with the β5 subunit), T‐L (β2 subunit), PGPH (β1 subunit), and BrAAP (β5 subunit), was observed, with the highest tested concentration inducing an almost complete inhibition. As reported in **Table** **1**, the three PVLs showed a subunit‐dependent specificity of inhibition. Considering the constitutive proteasome, C1 and C3 were particularly effective in inhibiting the T‐L component whereas C2 showed the highest inhibitory effect toward the ChT‐L. Globally, the catalytic components of the constitutive proteasome were more susceptible to the action of the three PVLs, showing lower IC_50_ values (down the nanomolar range) compared to the values obtained for the components of the immunoproteasome. The only exception was the BrAAP activity, whose inhibition was more evident in the immunoproteasome (see Table 1).

**Table 1:** IC_50_ values obtained from in vitro activity assays on isolated proteasomes

	IC_50_ [µM]		IC_50_ [µM]	
	ChT‐L		T‐L	
	Constitutive proteasome	Immunoproteasome	Constitutive proteasome	Immunoproteasome
C1	0.1196 ± 0.0121	0.1258 ± 0.0113	0.0130 ± 0.0021	0.2272 ± 0.0132^##^
C2	0.0162 ± 0.0012^**^	0.2379 ± 0.0167^*,##^	0.0874 ± 0.0079^**^	0.3344 ± 0.0289^*,#^
C3	0.1090 ± 0.0110	1.4520 ± 0.1530^**,##^	0.0568 ± 0.0023^*^	0.5876 ± 0.0476^*,##^

	PGPH		BrAAP	
	Constitutive proteasome	Immunoproteasome	Constitutive proteasome	Immunoproteasome
C1	0.0628 ± 0.0587	0.0418 ± 0.0038	0.2427 ± 0.0201	0.2179 ± 0.0196
C2	0.0858 ± 0.0078	0.1136 ± 0.0197^*,#^	0.2558 ± 0.0144	0.0613 ± 0.0057^*,#^
C3	0.1044 ± 0.0110	0.0727 ± 0.0057^*,#^	0.3024 ± 0.0278^*^	0.0884 ± 0.0070^*,#^

Asterisks refer to statistically significant differences obtained comparing C1 effects with C2/C3 effects on constitutive proteasomes or immunoproteasomes, respectively. Hashtags refer to statistically significant differences obtained comparing the effects of a single metabolite on the constitutive and immunoproteasome. ^*, #^ *p* < 0.05, ^**,##^ *p* < 0.01.

### 3.2 Effects of PVLs on Neuronal Cells Proteasomes

Next, SH‐SY5Y control and transfected cells were exposed to increasing concentration of PVLs (0‐10 µM) and viability was checked with the MTT assay. No cytotoxic effect was detected, with only a minor reduction in the number of APPmut viable cells upon 24 h exposure to 10 µM C2 (data not shown). Neuronal cells were then treated with 1 and 5 µM of each PVLs for 6 and 24 h.

**Figure 2:**
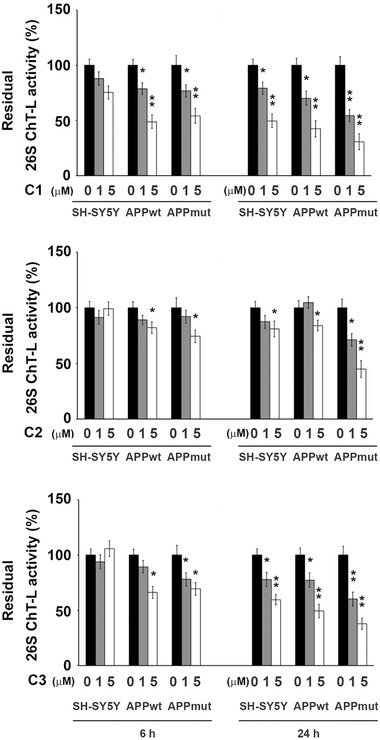
ChT‐L activity of the 26S proteasome measured in control and transfected SH‐SY5Y cells upon 6 and 24 h exposure to the three tested PVLs (C1, C2, and C3). Activities were measured using a fluorogenic peptide as a substrate as described in the Materials and methods section. Data are indicated as percentage versus untreated control/transfected cells (^*^ *p* < 0.05, ^**^ *p* < 0.01).

**Figure 3:**
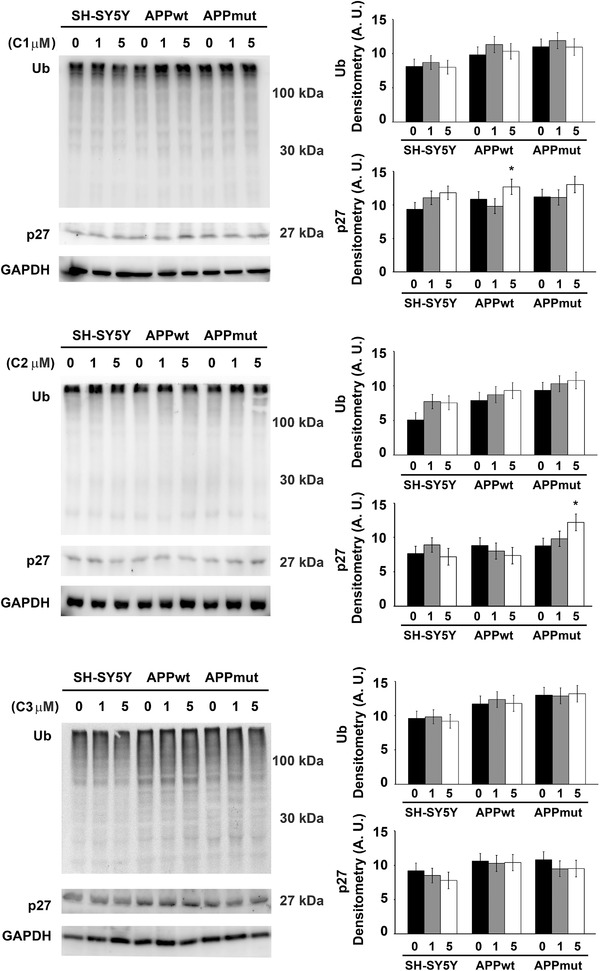
p27 and ubiquitin‐conjugates detected in control and transfected SH‐SY5Y cells upon 6 h exposure to PVLs. Representative immunoblots and densitometric analyses obtained from five separate experiments are shown (A.U. arbitrary units). Equal protein loading was verified by using an anti‐GAPDH antibody. Data points marked with an asterisk are statistically significant compared to the respective untreated cell line (^*^ *p* < 0.05).

The effects of the three compounds on the functionality of the proteasomal system, both the 20S core and the 26S proteasome, the latter consisting of a 20S core and one or two 19S regulatory particles, were then evaluated with fluorometric tests and immunoassays (**Figure** **2**). The inhibitory activity previously observed on isolated complexes was confirmed in the assays on cellular lysates. In detail, the metabolites induced the most remarkable effect upon 24‐h exposure displaying a concentration‐ and subunit‐dependent pattern of proteasome inhibition, with the BrAAP activity being the least affected (Sup. Figures 1–3, Supporting Information). Among the three tested compounds, the dihydroxylated metabolite C2 was the least efficient whereas C1 and C3 displayed a similar behavior strongly altering the functionality of the enzymatic complex, mainly the ChT‐L component of both the 20S and 26S proteasome (C1: 30% ChT‐L residual activity for both enzymes, C3: 50 and 60% ChT‐L residual activity for the 20S and 26S proteasome, respectively). Interestingly, the presence of the wt or mut APP sequence influenced the results and APPmut cells showed the highest extent of proteasomal inhibition. The inhibitory effect of the PVLs was confirmed by immunodetection of two proteasome cellular substrates, ubiquitin‐protein conjugates and p27. As shown in **Figures** **3** **and** **4**, no particular effect was observed upon 6 h exposure to C1, C2 and C3 whereas an evident increase in the levels of both Ub‐conjugates and p27 was obtained upon 24 h treatment, mainly in APPmut cells exposed to C1, confirming the data observed in the fluorescent assays. Together, these data show that these metabolites strongly affect proteasome functionality, selectively inhibiting its catalytic activity and favoring the accumulation of its related substrates.

**Figure 4:**
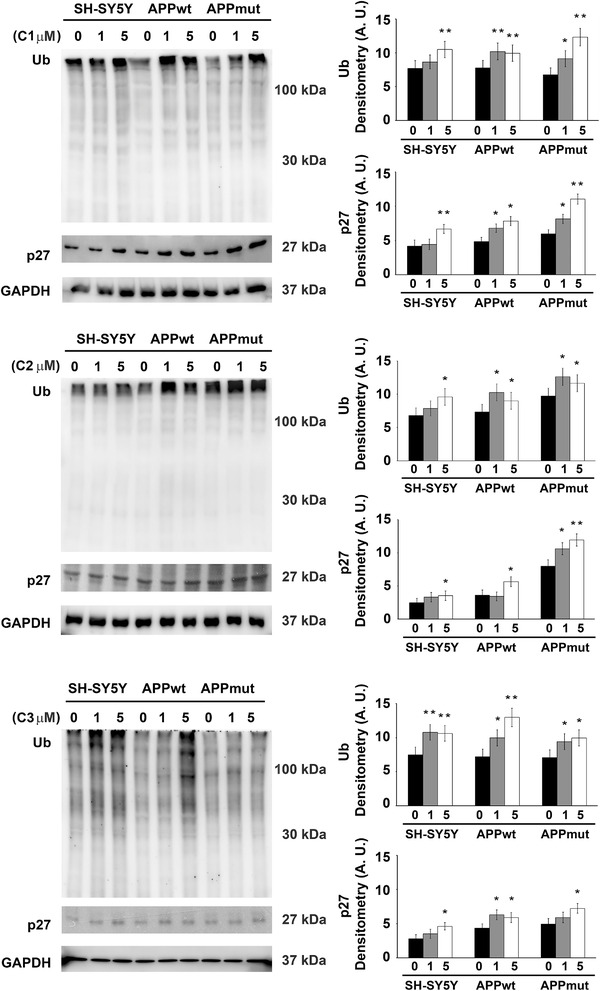
p27 and ubiquitin‐conjugates detected in SH‐SY5Y control and transfected cells upon 24 h exposure to PVLs. Representative immunoblots and densitometric analyses obtained from five separate experiments are shown (A.U. arbitrary units). Equal protein loading was verified by using an anti‐GAPDH antibody. Data points marked with an asterisk are statistically significant compared to the respective untreated cell line (^*^ *p* < 0.05, ^**^ *p* < 0.01).

In silico analyses were also conducted to dissect the mechanisms that mediate the interaction between the active site of the enzyme and the metabolites and to explain the observed inhibitory trend. The compounds of interest showed moderate binding affinities for proteasome catalytic subunits, with predicted equilibrium constants in the range 3–23 µM and a general conserved trend (C1>C2>C3). K_D_ values and energy contributions are summarized in **Table** **2**A and 2B.

**Table 2:** Computationally predicted affinities and energy contribution values for the complexes formed between human constitutive 20S proteasome catalytic subunits and C1, C2, and C3

A	Compound	K_D.pred_ [µM]	ΔG [kcal mol^–1^]	T. Energy [kcal mol^–1^]	I. Energy [kcal mol^–1^]	vdW Energy [kcal mol^–1^]	Electrostatic Energy [kcal mol^–1^]	Thr‐1‐Lactone distance [Å]
β1	C1	3.3	–7.475	–7.22	–23.971	–15.386	–8.585	6.8
	C2	19.09	–6.435	–6.754	–23.422	–10.742	–14.044	7.3
	C3	22.87	–6.328	–6.213	–26.784	–3.132	–24.939	6.9
β2	C1	7.12	–7.019	–19.119	–26.17	–13.22	–12.95	3.9
	C2	9.02	–6.879	–6.001	–24.086	–10.042	–14.044	4.5
	C3	12.86	–6.669	–6.931	–28.253	–3.314	–24.939	6.9
β5	C1	8.9	–6.887	–23.549	–32.468	–1.96	–30.508	6.9
	C2	12.43	–6.689	–7.364	–23.271	–2.321	–20.95	7.5
	C3	21.3	–6.37	–9.559	–30.029	4.666	–34.695	6.2

B	Compound	K_D.pred_ [µM]	ΔG [kcal mol^–1^]	T. Energy [kcal mol^–1^]	I. Energy [kcal mol^–1^]	vdW Energy [kcal mol^–1^]	Electrostatic Energy [kcal mol^–1^	Thr‐1‐Lactone distance [Å]
β1i	C1	7.959	–6.953	–19.898	–27.529	–13.506	–14.023	8.8
	C2	8.067	–6.945	–4.830	–21.318	–9.904	–11.414	6.1
	C3	22.87	–6.328	–5.438	–21.534	–0.924	–20.610	5.2
β2i	C1	9.487	–6.849	–22.519	–29.889	–8.034	–21.855	8.6
	C2	16.84	–6.509	–8.364	–24.732	–3.414	–21.318	8.6
	C3	16.87	–6.508	–12.007	–33.114	–1.473	–31.641	5.3
β5i	C1	5.973	–7.123	–25.270	–32.410	–12.876	–19.534	5.6
	C2	10.91	–6.766	–11.075	–28.657	–11.490	–17.167	5.9
	C3	10.99	–6.762	–13.963	–37.543	–8.997	–28.546	7.0

A, constitutive proteasome subunits; B, immunoproteasome subunits.

**Figure 5:**
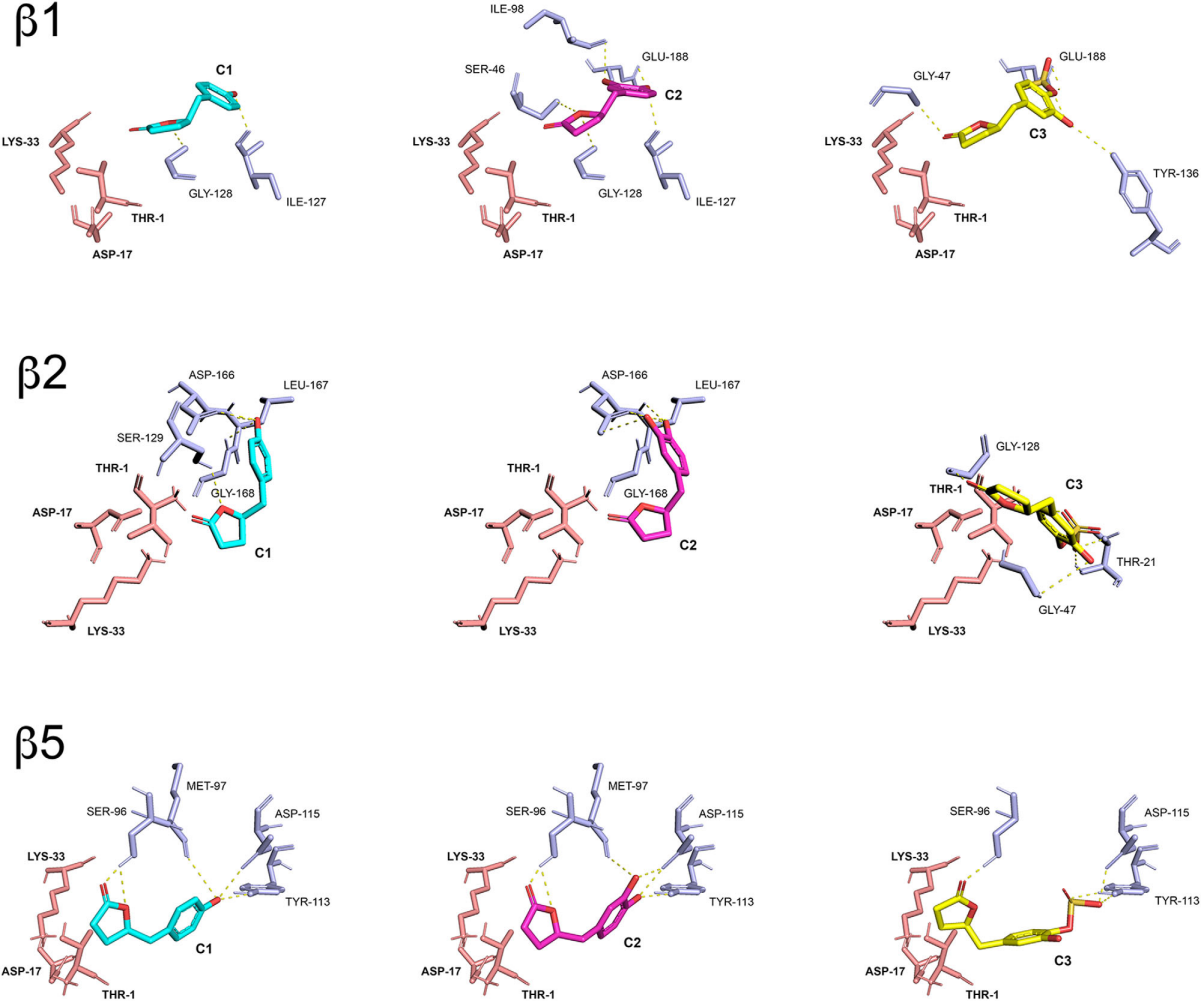
Comparative visualization of computed binding modes of PVLs C1, C2, and C3 to β1, β2, and β5 subunits of human constitutive 20S proteasome (pdb ID: 6rgq). Only the catalytic residues (Thr‐1, Asp‐17 and Lys‐33) and other residues in close proximity to the active site that are directly involved in the formation of H‐bonds are displayed as pink and light blue sticks, respectively. H‐bonds are indicated as yellow dashed solid lines.

Structurally, these molecules were predicted to establish a variable number of H‐bonds with amino acid residues in close proximity to the proteasome catalytic sites, with the carbonyl group of the lactone ring being always favorably positioned for a nucleophilic attack by the proteasome catalytic Thr‐1 residue, resulting in the opening of the γ‐lactone ring and acylation of the hydroxyl group, as expected for candidate proteasome inhibitors carrying lactone moieties^[^ ^30^ ^]^ (**Figures** **5** **and** **6**), and as supported by covalent docking analysis (see Figure S4, Supporting Information). Interestingly, the predicted distances between the hydroxyl group of Thr‐1 and the carbonyl group of the lactone and the consequent different tendency to form a covalent bond (more than the calculated binding affinity values) were in agreement with the general higher inhibitory effect toward constitutive proteasome observed in the in vitro studies, in particular with T‐L and BrAAP activities of constitutive and immuno‐proteasomes, respectively.

**Figure 6:**
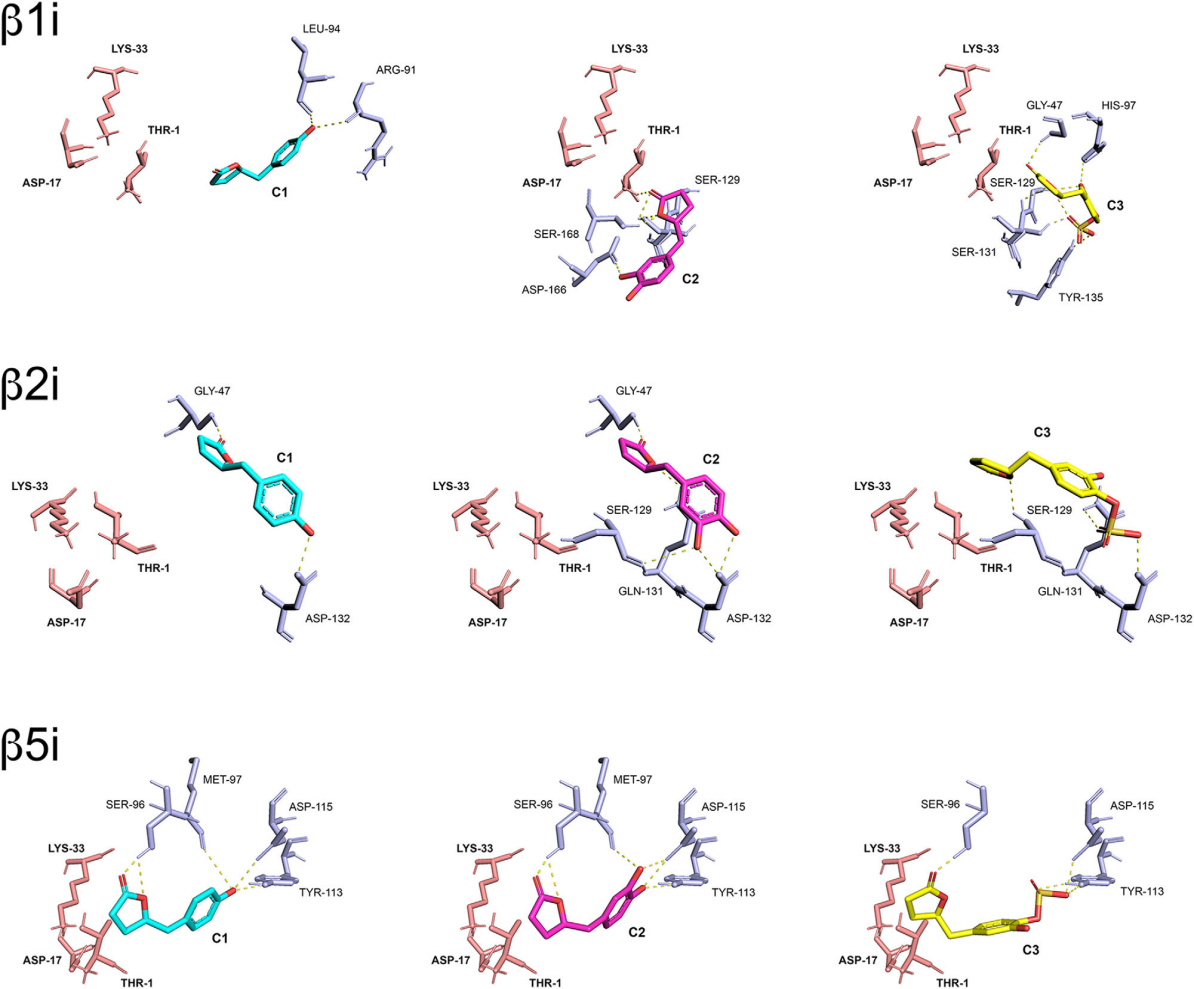
Comparative visualization of computed binding modes of PVLs C1, C2, and C3 to β1i, β2i, and β5i subunits of human immunoproteasome (pdb ID: 6e5b). Only the catalytic residues (Thr‐1, Asp‐17, and Lys‐33) and other residues in close proximity to the active site that are directly involved in the formation of H‐bonds are displayed as pink and light blue sticks, respectively. H‐bonds are indicated as yellow dashed solid lines.

### 3.3 Effects of PVLs on the Autophagic Pathway

**Figure 7:**
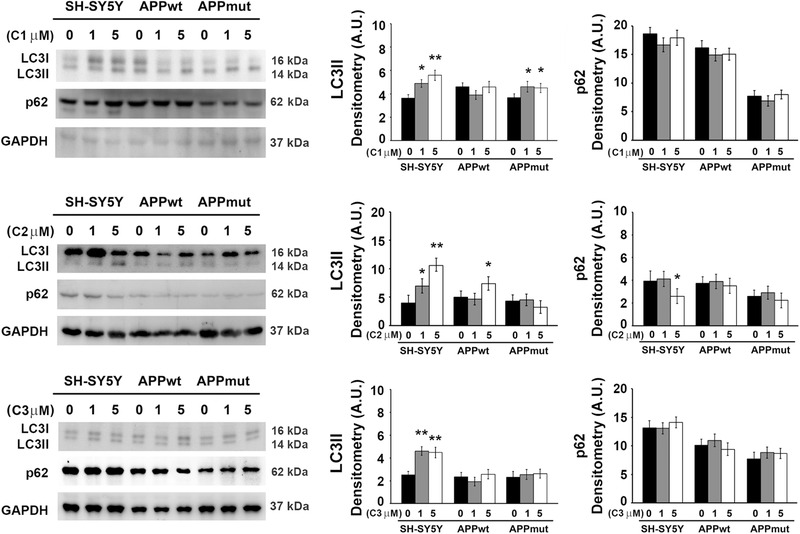
LC3II and p62 detected in SH‐SY5Y control and transfected cells upon 6 h exposure to PVLs. Representative immunoblots and densitometric analyses obtained from five separate experiments are shown (A.U. arbitrary units). Equal protein loading was verified by using an anti‐GAPDH antibody. Data points marked with an asterisk are statistically significant compared to the respective untreated cell line (^*^ *p* < 0.05, ^**^ *p* < 0.01).

**Figure 8:**
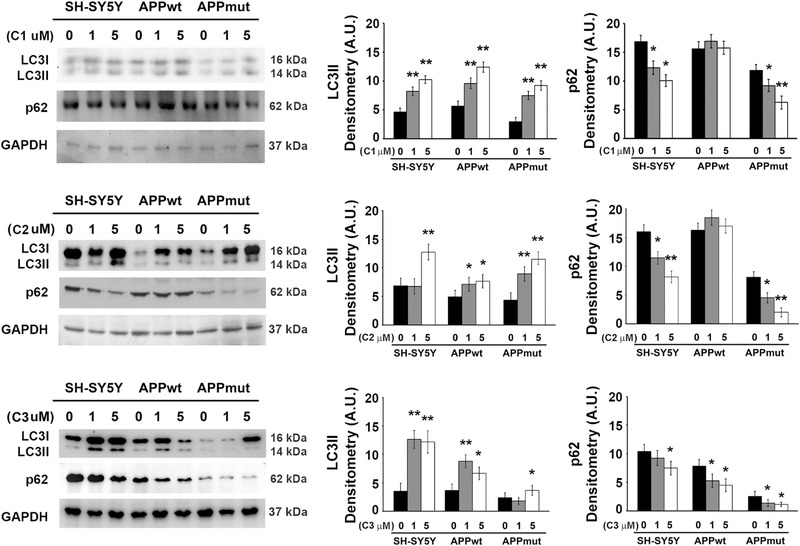
LC3II and p62 detected in SH‐SY5Y control and transfected cells upon 24 h exposure to valerolactones. Representative immunoblots and densitometric analyses obtained from five separate experiments are shown (A.U. arbitrary units). Equal protein loading was verified by using an anti‐GAPDH antibody. Data points marked with an asterisk are statistically significant compared to the respective untreated cell line (^*^ *p* < 0.05, ^**^ *p* < 0.01).

**Figure 9:**
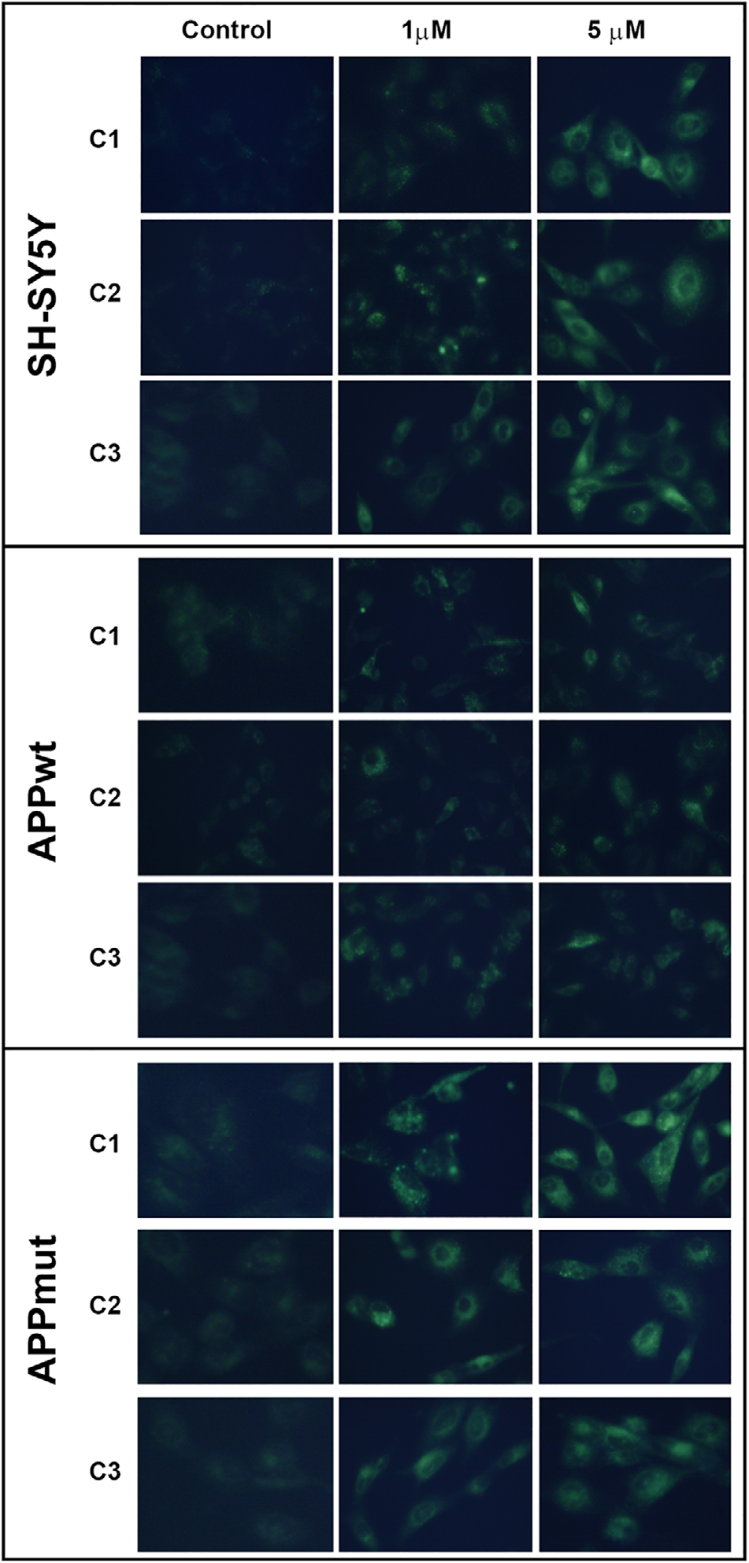
MDC staining of induced autophagic vacuoles in neuronal cells treated with C1, C2, and C3 (Control, 1 and 5 µM). Cells were treated with PVLs for 24 h and then incubated with MDC dye as indicated in the Materials and methods section.

Together with the proteasome, autophagy represents a major quality control system responsible for the maintenance of cellular homeostasis. The effect on autophagy of the three PVLs was evaluated measuring the expression of proteins involved in this pathway, such as LC3II, the lipidated form of LC3 that localizes in autophagosomal membranes, and p62, a substrate of autophagy that accumulates in cells when autophagy is inhibited.^[^ ^3^ ^]^ Upon 6 h treatment with the three metabolites (1 and 5 µM), SH‐SY5Y control cells showed increased levels of LC3II but almost no change in the expression of the p62 protein, suggesting the activation of the early steps of the autophagic pathway in this cell line (**Figure** **7**). A minor effect was observed at this time point in the two transfected clones, as LC3II increased in APPwt and APPmut cells upon exposure to C2 and C1, respectively and no variations in p62 amounts were detectable. Interestingly, the 24 h treatment markedly modified the levels of both proteins not only in untransfected cells but also in APPmut cells, demonstrating the complete activation of autophagy with a significant downregulation of the p62 protein (**Figure** **8**). APPwt cells were the least sensible to the action of the compounds. In details, upon long‐term exposure to the three PVLs, this clone showed increased levels of LC3II but only 5‐(3ʹ‐hydroxyphenyl)‐γ‐valerolactone‐4ʹ‐sulfate (C3) favored p62 degradation, suggesting the complete activation of autophagy. To further confirm data on the activation of the autophagic pathway, we measured the amounts of autophagic vacuoles staining PVLs‐treated cells with the autofluorescent dye MDC.^[^ ^31^ ^]^ **Figure** **9** shows the increased number of autophagosomes in the three cell lines treated for a period of 24 h with the PVLs of interest. Increased numbers of autophagic vacuoles were detected in all the treated cell lines, but they were particularly evident in normal SH‐SY5Y and APPmut cells.

### 3.4 PVLs Effect on Cathepsin B Activity

**Figure 10:**
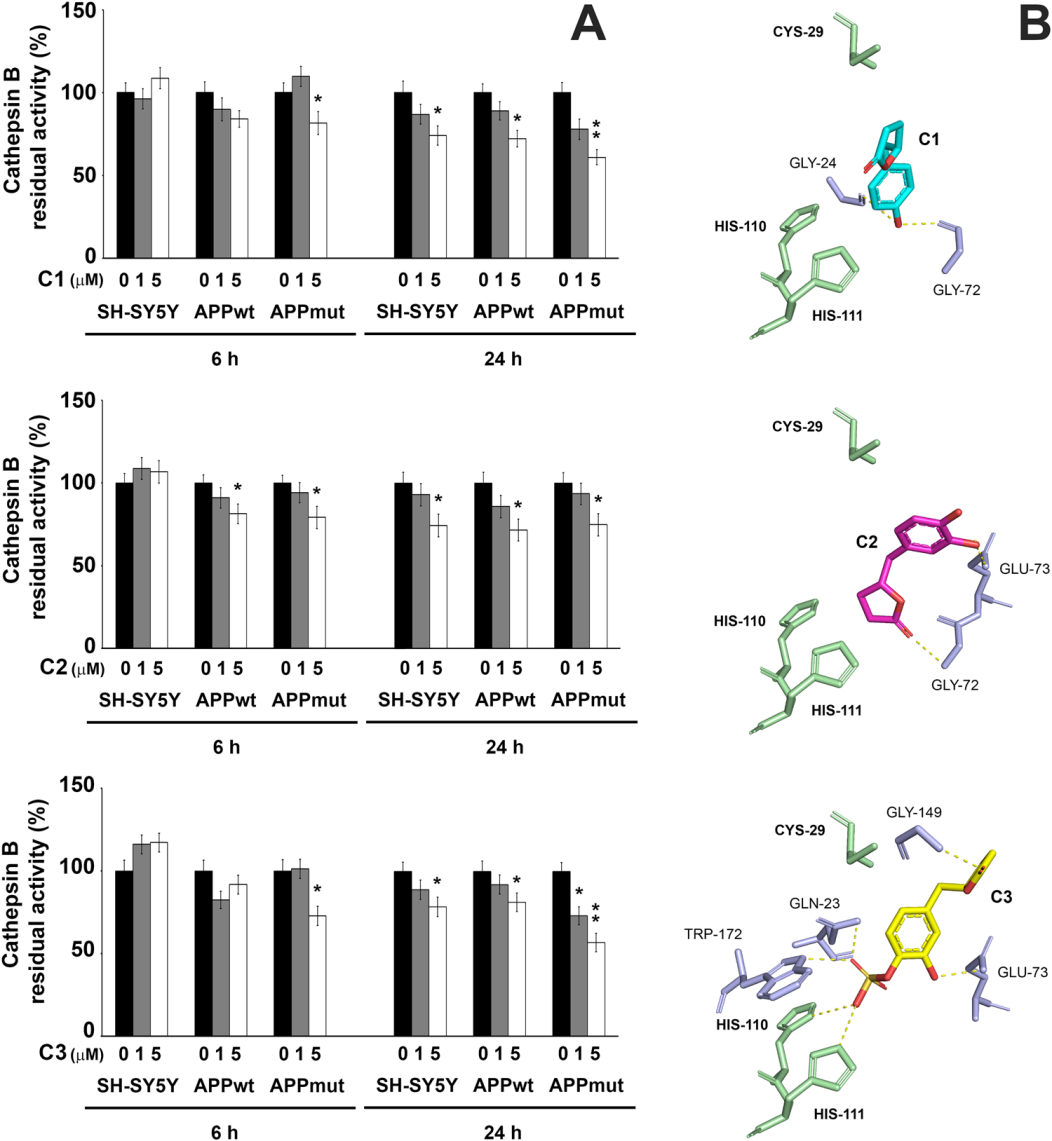
A) Cathepsin B activity measured in SH‐SY5Y control and transfected cells upon 6 and 24‐h exposure to PVLs. Activity was measured using a fluorogenic peptide as substrate as described in the Materials and Methods section. Data are indicated as percentage versus untreated control/transfected cells (^*^ *p* < 0.05, ^**^ *p* < 0.01). B) Comparative visualization of computed binding modes of valerolactones C1, C2, and C3 to human cathepsin B (pdb ID: 1csb). Only the residues involved in the catalysis (Cys‐29, His‐110, and His‐111) and other residues in close proximity to the active site that are directly involved in the formation of H‐bonds are displayed as light green and light blue sticks, respectively. H‐bonds are indicated as yellow dashed solid lines.

Cathepsin B is a lysosomal cysteine protease that was shown to be upregulated in AD subjects and to play a role in plaques formation and consequent behavioral deficits and neuropathology of AD.^[^ ^32^ ^]^ We previously characterized the three cell lines in terms of cathepsin B activity, highlighting that it is strongly upregulated in APPmut cells, with no differences between APPwt cells and control neuronal cells.^[^ ^24^ ^]^ Growing evidence suggests that cathepsin B inhibition is able to reduce Aβ levels.^[^ ^33^, ^34^, ^35^ ^]^ Cathepsin B functionality was measured in cell lysates after the exposure to the three PVLs. Upon 6 h treatment, only APPmut cells showed an inhibited cathepsin B activity (20% inhibition upon 5 µM C1 and C2 treatment and 30% inhibition upon 5 µM C3 treatment), whereas after 24 h, a decreased functionality of the enzyme was observed also in control and APPwt cells (**Figure** **10**, panel A). Similarly, a computational predictive study was conducted to characterize the interaction between C1, C2 and C3 and cathepsin B. This in silico analysis indicated interactions with moderate affinities (K_D_ in the range 1–5 µM, with the same trend displayed for proteasome (**Table** **3** III)). Structurally, the valerolactones were predicted to establish H‐bonds with amino acid residues of the active site cleft of the enzyme, the resulting binding pose being likely to prevent substrate access to the catalytic Cys‐29,^[^ ^36^ ^]^ consistently with our experimental evidence. Again, the lactone carbonyl was favorably positioned for a nucleophilic attack by the thiol group of Cys‐29 (Figure 10, panel B), in line with the possible acylation of cysteine (Figure S5).^[^ ^37^ ^]^

**Table 3:** Predictive affinities and energy contribution values for the complexes formed between human cathepsin B and C1, C2, and C3

Compound	K_D,pred_ [µM]	ΔG [kcal mol^–1^]	T. Energy [kcal mol^–1^]	I. Energy [kcal mol^–1^]	vdW Energy [kcal mol^–1^]	Electrostatic Energy [kcal mol^–1^]	Cys‐29‐Lactone distance [Å]
C1	1.61	–7.899	–10.161	–26.132	–20.005	–6.127	8.8
C2	4.26	–7.323	–11.223	–27.287	–12.506	–14.781	6.1
C3	5.53	–7.169	–25.500	–35.483	–15.572	–19.911	5.2

### 3.5 Amyloid‐β(1‐42) Levels Upon Exposure to PVLs

As previously reported, APPwt and APPmut clones show comparable APP expression levels, higher than control SH‐SY5Y cells, and generate and release elevated amounts of Aβ(1‐42) peptide in their culture media.^[^ ^24^ ^]^ Furthermore, APPmut cells produced and released significant higher levels of Aβ(1‐42) peptide than APPwt cells.^[^ ^38^ ^]^ The levels of this peptide were detected both in the medium and cellular extracts of treated control and transfected SH‐SY5Y cells using an ELISA kit.

**Figure 11:**
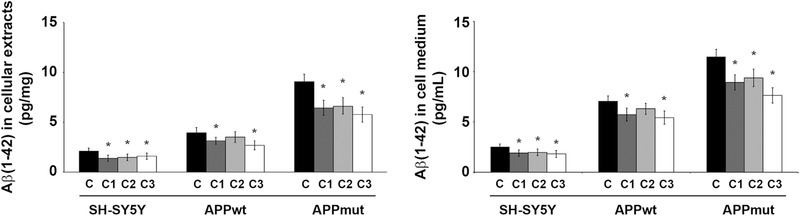
Levels of Aβ(1‐42) measured in cellular extracts and cell medium upon 24 h treatment with C1, C2, and C3 (5 µM). Protein levels were measured using an ELISA kit as described in the Materials and Methods section. Data are indicated as percentage versus untreated control/transfected cells (^*^ *p* < 0.05, ^**^ *p* < 0.01).

PVLs treatment significantly reduced the amounts of intracellular and extracellular Aβ(1‐42) with comparable final effects in the two tested samples. In detail, C3 was the most effective in reducing the amount of the toxic peptide in APPmut cells (35% reduction both in cell extracts and cell medium) (**Figure** **11**). The monohydroxylated metabolite (C1) exerted similar effects on the three cell lines whereas C2, the less active among the three metabolites, did not induce any change in Aβ(1‐42) levels in APPwt cells. Interestingly, these data on the amount of intracellular and released amyloid peptide reflect the activation of the autophagic pathway induced by the compounds with the exception of C1 and its effect on APPwt cells. In fact, although it was not able to completely activate autophagy in this cell line, it significantly reduced the amounts of Aβ(1‐42) peptide.

## 4 Discussion

Defective proteolytic pathways and accumulation of amyloid peptides in senile plaques characterize AD neuropathology and contribute to altered cognition and memory. A proper modulation of intracellular proteolysis could therefore help in ameliorating AD condition. On this regard, dietary polyphenols and their derivatives, besides the antioxidant and anti‐inflammatory activity, were reported to regulate proteolysis and to act as anti‐amyloid agents, making them interesting candidates in preventative strategies against AD.^[^ ^39^ ^]^ An increasing number of studies is now dissecting the biological properties of PVLs, products of intestinal microbial metabolism of flavan‐3‐ols, that constitute a rich portion of phenolic metabolites in the circulation of subjects exposed to the widely spread dietary sources of this subclass of compounds.^[^ ^8^ ^]^

In this study, we investigated the ability of three PVLs to modulate proteolysis in SH‐SY5Y neuronal cells and to counteract the production and release of the toxic Aβ(1‐42) peptide, at levels readily achievable in body fluids upon moderate consumption of flavonoid‐rich foods or beverages.^[^ ^40^, ^41^ ^]^ In details, two microbial metabolites with different hydroxylation patterns, the 5‐(4ʹ‐hydroxyphenyl)‐γ‐valerolactone and the 5‐(3ʹ,4ʹ‐dihydroxyphenyl)‐γ‐valerolactone, and the conjugated derivative 5‐(3ʹ‐hydroxyphenyl)‐γ‐valerolactone‐4ʹ‐sulfate, were tested.

The three compounds similarly affected the functionality of isolated 20S proteasomes, both the constitutive and the immunoproteasome, inducing an almost complete inhibition at the highest doses. They were particularly effective in inhibiting the catalytic subunits of the constitutive complex, with the exception of the BrAAP subunit that showed the highest inhibition in the immunoproteasome. In silico computational studies better clarified the mechanisms behind the observed inhibitory trend, underlining the central role of the lactone moiety, produced by the action of intestinal bacteria that open the C‐ring of the flavan‐3‐ol precursor and convert it into the PVL.^[^ ^42^, ^43^ ^]^ This group is likely to undergo a nucleophilic attack by the N‐terminal threonine of the active site of proteasomes and covalently inhibits the enzyme, like previously reported for proteasome inhibitors carrying the same functional group.^[^ ^30^ ^]^ This newly generated lactone group strongly increases the inhibitory potency of PVLs with respect to parental catechins such as (−)‐epicatechin and EGCG.^[^ ^44^ ^]^

We then treated human SH‐SY5Y neuroblastoma cells, control and APPwt/APPmut transfected cells, with the three metabolites and evaluated their effects on cellular proteolysis. Previous studies from our laboratory indicated the presence of a less functional proteasome in APP transfected cells, mainly the APPmut clone.^[^ ^24^ ^]^ PVLs inhibited the 20S and 26S complexes in a time‐ and subunit‐dependent manner, with the monohydroxylated form (C1, 5‐(4ʹ‐hydroxyphenyl)‐γ‐valerolactone) and the sulfated derivative (C3, 5‐(3ʹ‐hydroxyphenyl)‐γ‐valerolactone‐4ʹ‐sulfate) resulting the most effective. APPmut cells showed the highest degree of proteasome inhibition likely due to an already compromised enzymatic complex, as a consequence of the higher amounts of amyloid peptides released. These results indicate that PVLs can act like proteasome modulators as widely reported for their flavan‐3‐ol precursors and other polyphenols.^[^ ^45^, ^46^, ^47^, ^48^ ^]^ While comparisons with other compounds may be biased by the different experimental settings, the prospects of these PVLs have been demonstrated. Further works should be designed to understand the relationship among these microbial‐derived phenolic metabolites and phase II‐conjugated flavan‐3‐ols able to cross the BBB, identifying the most active metabolites upon flavan‐3‐ol consumption, and investigating their synergistic, additive, and/or antagonistic effects occurring in presence of multiple metabolites.

The coordinated action of proteolytic systems is fundamental for protein quality control and for the maintenance of cellular homeostasis, especially in conditions where protein aggregates easily tend to accumulate. Numerous studies described a marked alteration of both the proteasome and autophagy in neurodegenerative diseases, including Parkinson's disease, Huntington's disease, amyotrophic lateral sclerosis, frontotemporal dementia, and AD, with these defects favoring aggregates‐mediated toxicity.^[^ ^49^, ^50^, ^51^ ^]^ Considering the complex and the dynamic nature of the autophagic pathway,^[^^]^ we monitored several markers associated with different steps of this process. Our data show that PVLs treatment upregulated autophagy in the three cell lines, mainly in untransfected and APPmut cells, as shown by the increase in autophagosomes formation and the simultaneous reduction of p62 levels. The complete activation of the autophagic process in these two cell lines was observed upon 24 h exposure whereas only SH‐SY5Y control cells showed an increased amount of LC3II upon the short time treatment, suggesting an earlier response of untransfected cells to the treatment. These findings indicate that the presence of the wild‐type or mutated sequence of the APP influences the final effect of the metabolites on this proteolytic pathway. Among the tested compounds, only 5‐(3ʹ‐hydroxyphenyl)‐γ‐valerolactone‐4ʹ‐sulfate (C3) was able to trigger complete autophagy in APPwt cells.

We then dissected the effects of the three metabolites on cathepsin B activity. This enzyme was associated with the amyloidogenic APP processing and the consequent release of amyloid peptides^[^ ^35^ ^]^ and we previously found that the expression of the V717G mutated sequence of APP in SH‐SY5Y cells strongly elevated the activity of the hydrolase.^[^ ^24^ ^]^ Our in silico and experimental data indicate that these PVLs can effectively inhibit the activity of this enzyme preventing the substrate access to the catalytic Cys‐29 residue. The slight differences observed in cathepsin B inhibition between control and transfected cells suggest that the presence of the wt or mut APP sequence does not significantly alter the final effect of these metabolites on the protease.

Considering the ability of PVLs to modulate cellular proteolytic systems involved in Aβ generation and processing, we investigated possible effects on the production and release of the Aβ(1‐42) peptide. Autophagy activation in response to inhibition of the proteasome is frequently observed in cells and it is considered as a compensatory protective mechanism to guarantee the elimination of protein aggregates and alleviate associated proteotoxic stress.^[^ ^52^, ^53^, ^54^ ^]^ This crosstalk between protein degradative pathways assumes a central role in neurodegenerations, including AD, and it was demonstrated to help neurons getting rid of detrimental Aβ(1‐42) peptides.^[^ ^33^, ^55^, ^56^ ^]^ In addition, it was demonstrated that cathepsin B exhibits β‐secretase activity in secretory vesicles of neuronal chromaffin cells and that its inhibitors can lower amyloid levels produced from APP in guinea pigs, suggesting that a reduction in cathepsin B functionality can ameliorate AD condition.^[^ ^32^, ^34^, ^35^ ^]^ In line with these observations, the three tested PVLs diminished the amounts of Aβ(1‐42) in the medium and lysates of neuronal cells with the only exception of C2 that did not modify the levels of the toxic peptide in APPwt cells. On this regard, metabolites C1 and C2 exhibited a comparable effect in this cell line showing a limited activity in upregulating autophagy and inducing a significant inhibition of cathepsin B functionality. It is therefore reasonable to think that, beyond cathepsin B inhibition, 5‐(4ʹ‐hydroxyphenyl)‐γ‐valerolactone (C1) exerts its effect against amyloid peptides through additional mechanisms, likely involving other enzymes responsible for APP processing and amyloid production.

These results on the anti‐amyloid and neuroprotective effects of the considered metabolites are in line with previous findings on PVLs ability to detoxify amyloid‐β oligomers and prevent memory impairment in AD.^[^ ^57^ ^]^ Also relevant in this context are data showing that these metabolites can cross the blood‐brain barrier, reach the brain parenchyma and promote neurogenesis in the brain.^[^ ^11^, ^12^ ^]^ Together, these data further confirm the beneficial role of a diet rich in catechins and proanthocyanidins, whose metabolites can successfully reduce the presence of molecular markers associated with the onset and progression of neurodegeneration.

In conclusion, our findings provide, for the first time, evidence for the neuroprotective activity of PVLs, microbiota‐derived metabolites of flavan‐3‐ols, associated with the modulation of intracellular proteolytic systems and suggest their use in preventative strategies against AD. Future experiments should be performed to enrich the evidence gathered so far. In this regard, a better understanding of the journey of flavan‐3‐ol metabolites into the brain, addressing transportation mechanisms through the BBB, and taking into account their effects in the framework of animal models of AD may serve to provide effective, tailored dietary recommendations.

## Conflict of Interest

The authors declare no conflict of interest.

## Author Contributions

A.M.E., conceived and designed the study, revised the manuscript; C.V., designed and performed the experiments and wrote the paper; M.C., performed and analyzed the in silico studies and revised the manuscript; M.A. analyzed the in silico studies and revised the manuscript; Y.Z. performed experiments with neuronal cells; L.B., C.G., P.M., and D.D.R. assisted in data analysis and revised the manuscript.

## Supporting information

Supporting Information.

## Data Availability

Data available on request from the authors.
