# Supplementary material for: Flavan‐3‐ol Microbial Metabolites Modulate Proteolysis in Neuronal Cells Reducing Amyloid‐beta (1‐42) Levels
Source: Mol Nutr Food Res. 2021 Aug 7;65(18):2100380. doi: 10.1002/mnfr.202100380 (PMC9285603; doi:10.1002/mnfr.202100380)
Supplement: Supplementary file 1 — Supporting Information. [file MNFR-65-0-s001.docx]

**Supporting information**

**
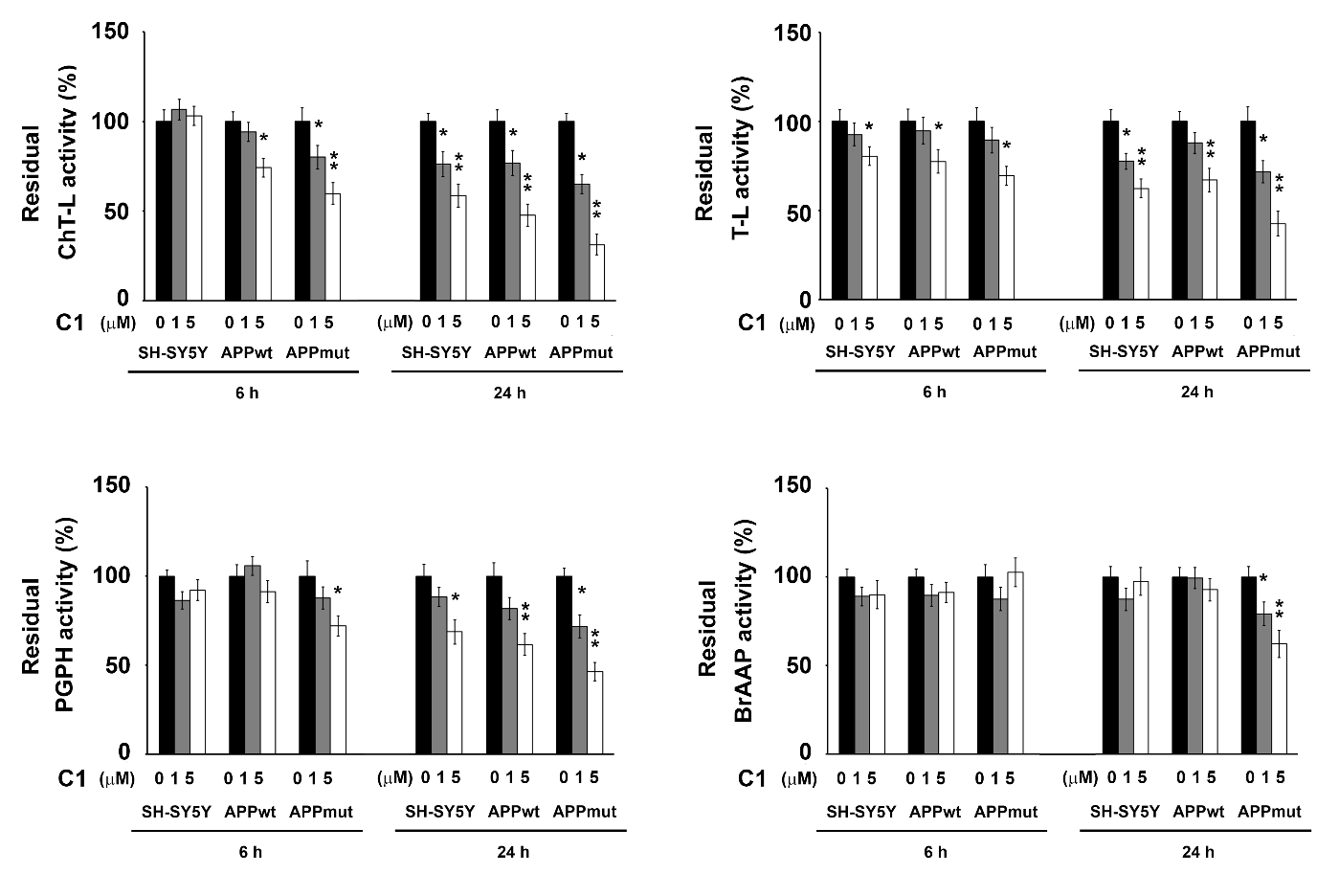
**

Figure S1. Proteasome ChT-L, T-L, PGPH and BrAAP activities measured in control and transfected SH-SY5Y cells upon 6 and 24 h exposure to C1. Activities were measured using a fluorogenic peptide as a substrate as described in the Materials and methods section. Data are indicated as percentage vs. untreated control/transfected cells (*p<0.05, **p<0.01).

**
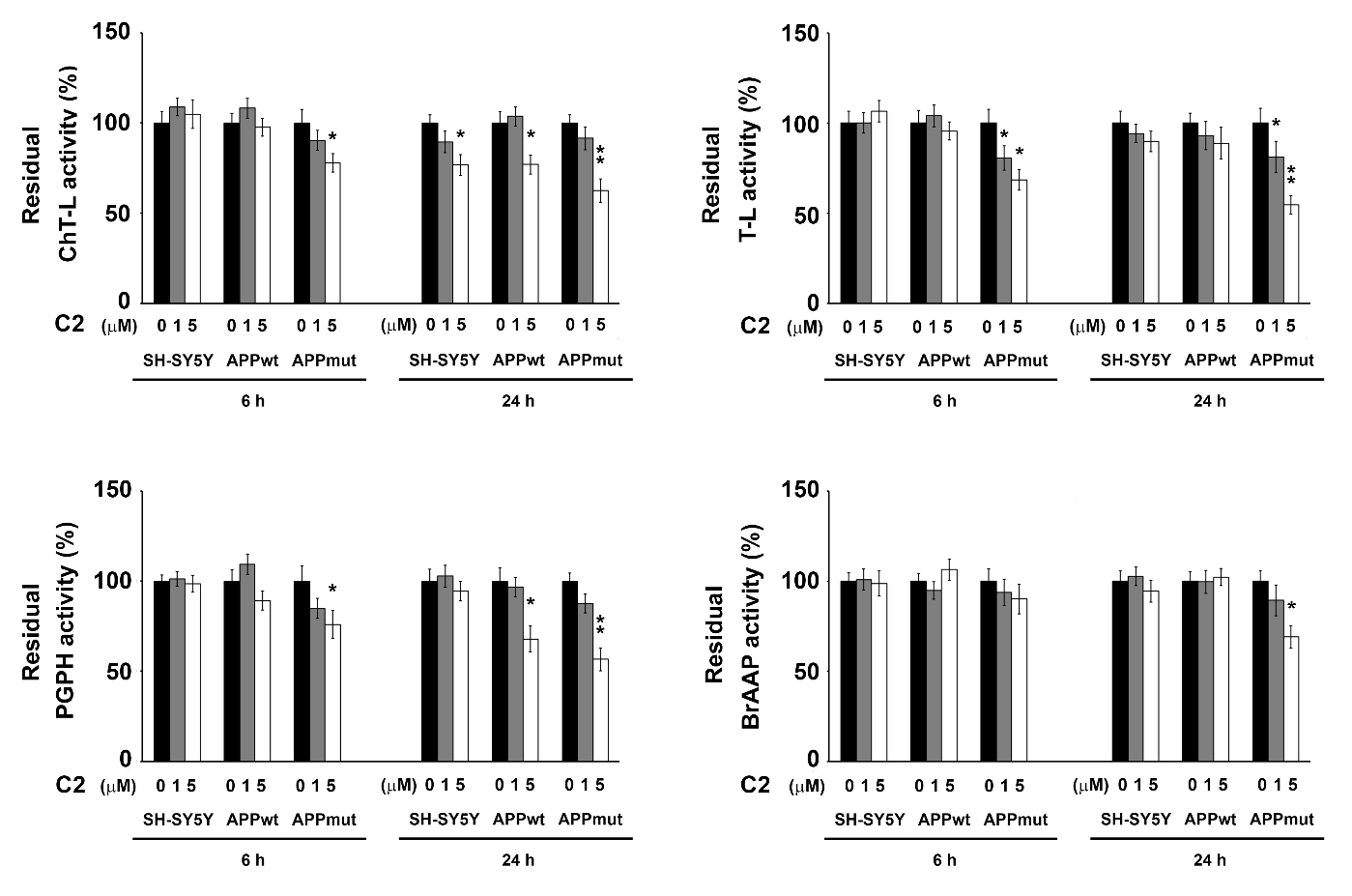
**

Figure S2. Proteasome ChT-L, T-L, PGPH and BrAAP activities measured in control and transfected SH-SY5Y cells upon 6 and 24 h exposure to C2. Activities were measured using a fluorogenic peptide as a substrate as described in the Materials and methods section. Data are indicated as percentage vs. untreated control/transfected cells (*p<0.05, **p<0.01).

**
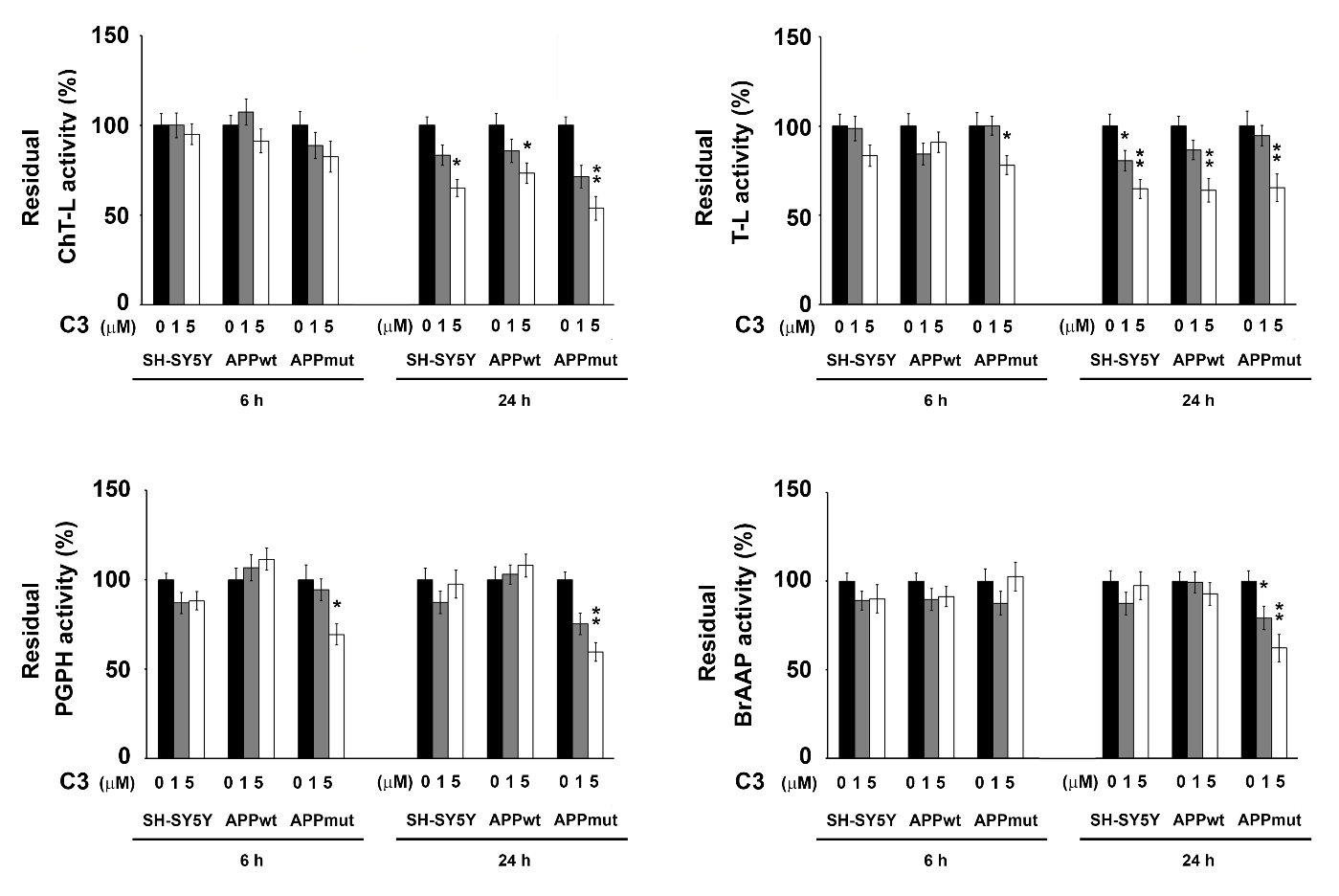
**

Figure S3. Proteasome ChT-L, T-L, PGPH and BrAAP activities measured in control and transfected SH-SY5Y cells upon 6 and 24 h exposure to C3. Activities were measured using a fluorogenic peptide as a substrate as described in the Materials and methods section. Data are indicated as percentage vs. untreated control/transfected cells (*p<0.05, **p<0.01).

**
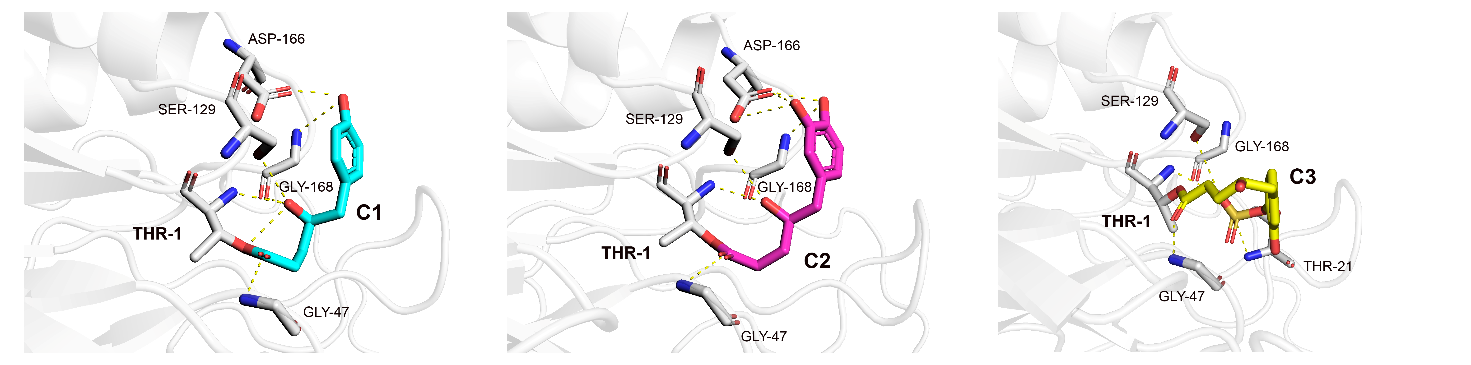
**

Figure S4. Binding models of C1, C2 and C3 bound to Thr-1 catalytic residue of constitutive 20S proteasome. Covalent docking was performed using Dockovalent (Covalent docking of large libraries for the discovery of chemical probes. Nir London, Rand M Miller, Shyam Krishnan, Kenji Uchida, John J Irwin, Oliv Eidam, Lucie Gibold, Peter Cimermančič, Richard Bonnet, Brian K Shoichet & Jack Taunton Nature Chemical Biology volume 10, pages 1066–1072(2014)). Amino acids involved in the formation of covalent and H-bonds are highlighted as sticks.


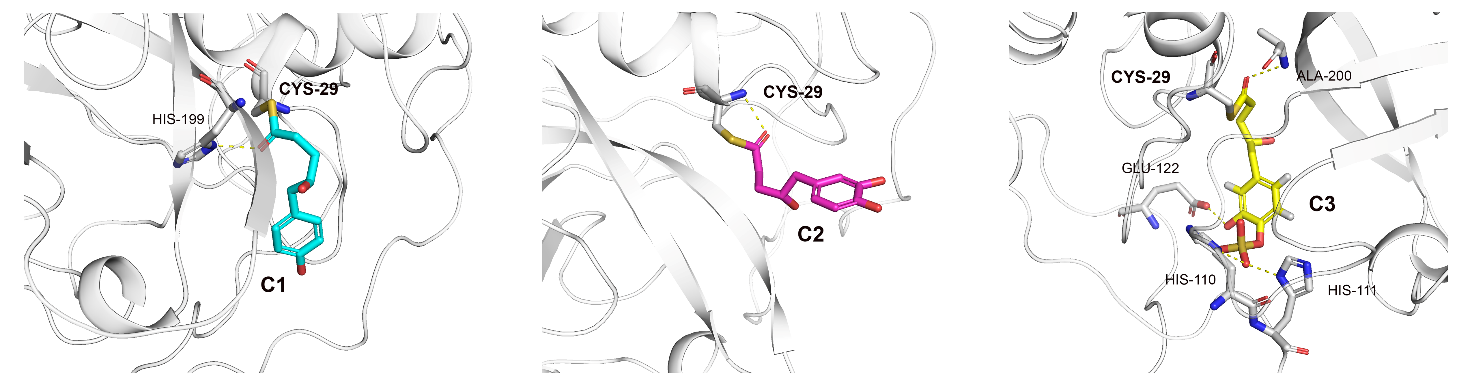


Figure S5. Binding models of C1, C2 and C3 bound to Cys-29 catalytic residue of cathepsin B. Covalent docking was performed using Dockovalent (Covalent docking of large libraries for the discovery of chemical probes. Nir London, Rand M Miller, Shyam Krishnan, Kenji Uchida, John J Irwin, Oliv Eidam, Lucie Gibold, Peter Cimermančič, Richard Bonnet, Brian K Shoichet & Jack Taunton Nature Chemical Biology volume 10, pages 1066–1072(2014)). Amino acids involved in the formation of covalent and H-bonds are highlighted as sticks.
